# Insights into the roles of inositol hexakisphosphate kinase 1 (IP6K1) in mammalian cellular processes

**DOI:** 10.1016/j.jbc.2024.107116

**Published:** 2024-02-24

**Authors:** Mohamed Chakkour, Miriam L. Greenberg

**Affiliations:** Department of Biological Sciences, Wayne State University, Detroit, Michigan, USA

**Keywords:** 5-IP7, inositol phosphate, inositol pyrophosphates, gene expression, metabolism, cell migration, phosphorylation

## Abstract

Inositol phosphates and their metabolites play a significant role in several biochemical pathways, gene expression regulation, and phosphate homeostasis. Among the different inositol phosphates, inositol hexakisphosphate (IP6) is a substrate of inositol hexakisphosphate kinases (IP6Ks), which phosphorylate one or more of the IP6 phosphate groups. Pyrophosphorylation of IP6 leads to the formation of inositol pyrophosphates, high-energy signaling molecules that mediate physiological processes through their ability to modify target protein activities, either by directly binding to their target protein or by pyrophosphorylating protein serine residues. 5-diphosphoinositol pentakisphosphate, the most abundant inositol pyrophosphate in mammals, has been extensively studied and found to be significantly involved in a wide range of physiological processes. Three IP6K (IP6K1, IP6K2, and IP6K3) isoforms regulate IP7 synthesis in mammals. Here, we summarize our current understanding of IP6K1's roles in cytoskeletal remodeling, trafficking, cellular migration, metabolism, gene expression, DNA repair, and immunity. We also briefly discuss current gaps in knowledge, highlighting the need for further investigation.

Maintaining phosphate homeostasis is vital for growth and survival, as nearly all biochemical processes depend on phosphate. In mammals, a sophisticated hormonal regulatory network, spanning the intestines, bones, and kidneys, tightly controls the concentration of serum phosphate (1). Inositol phosphates play a key role in phosphate homeostasis (2) and have been recognized as crucial signaling molecules that participate in a wide array of cellular processes, from growth regulation to apoptosis (3). Among the inositol phosphates, inositol hexakisphosphate (IP6), a molecule consisting of six phosphate groups attached to an inositol ring, is especially noteworthy for phosphate storage, cellular signaling, and immune modulation. IP6 is the substrate of inositol hexakisphosphate kinases (IP6Ks), which phosphorylate one or more of the IP6 phosphate groups, leading to the formation of several inositol pyrophosphates (IPs). Among these, 5-diphosphoinositol pentakisphosphate (5-IP7) is the most abundant IP in mammals (Fig. 1) (4, 5). IPs are signaling molecules that possess a high-energy phosphoanhydride bond. They engage in a wide variety of different physiological processes, either by binding specific proteins or by transferring their β phosphate to prephosphorylated serine residues, resulting in serine pyrophosphorylation (6, 7). Particularly, 5-IP7 can directly, without requiring a protein kinase, phosphorylate its target proteins in a Mg^2+^-dependent mechanism (8). However, prior to their pyrophosphorylation by 5-IP7, serine-rich residues in target proteins must be primed with ATP-dependent phosphorylation (Fig. 1). This process of pyrophosphorylation is a novel modification that grants a distinctive mode of signaling to proteins (9). Interestingly, the role of IPs as essential second messengers in several signaling pathways seems to outweigh the role of inositol phosphates (3).

**Figure 1:**
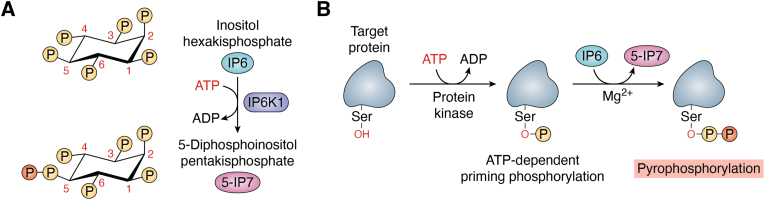
**The pyrophosphorylation role of IP6K1 and 5-IP7.***A*, inositol hexakisphosphate kinase 1 (IP6K1) phosphorylates inositol hexakisphosphate (IP6) on phosphorylated carbon number 5. This ATP-dependent reaction, termed pyrophosphorylation, is characterized by the addition of a phosphate group to a pre-existing phosphorylation mark (P). The product of this reaction is the inositol pyrophosphate 5-diphosphoinositol pentakisphosphate (5-IP7). *B*, 5-IP7 pyrophosphorylates its target proteins independently, without the need for ATP or protein kinases. Target proteins must first be primed by the action of a protein kinase, which phosphorylates a serine residue (Ser) within the target protein. This ATP-dependent phosphorylation tags the target protein for further pyrophosphorylation by 5-IP7 in the presence of Mg^2+^.

IP6Ks are present in all eukaryotic organisms. In lower organisms, such as yeast, slime molds, and *Drosophila*, a single IP6K is typically found. In mammals, three IP6K isoforms exist—IP6K1, IP6K2 and IP6K3. All three isoforms are encoded by distinct genes and exhibit similarities in their C-terminal domains and differences in their N-terminal domains (10, 11). Among them, IP6K1 and IP6K2 are expressed in multiple tissues (4), whereas IP6K3 exhibits significant expression primarily in the cerebellum (12).

Several studies have shown that IP6Ks impact numerous cellular and physiological processes in mammals, including cell growth, apoptosis, DNA repair, and cellular stress responses (5, 13). Several genetic knockout experiments targeting IP6K isoforms have been performed in both *in vitro* and *in vivo* systems. These investigations have revealed noticeable variations in phenotypes among the three family members, despite their involvement in the catalysis of the same reaction. For instance, IP6K1 was shown to be involved in DNA repair, chromatin modification, and glucose/insulin homeostasis. IP6K2 is essential in promoting cell death, tumor growth, and metastasis. IP6K3 is involved in neuronal development and metabolic regulation (3, 14).

To the best of our knowledge, there are no recent reviews focused on the role of IP6K1 in mammalian cellular signaling. This provides a comprehensive summary of the roles of IP6K1 in different mammalian cellular metabolic pathways. The following sections elaborate on how, over the past 15 years, research has shown the involvement of IP6K1 in metabolism, modulation of immune cells, gene expression, DNA repair, cellular migration, and reorganization of the cytoskeleton.

## Trafficking, migration, and cytoskeletal remodeling

**Figure 2:**
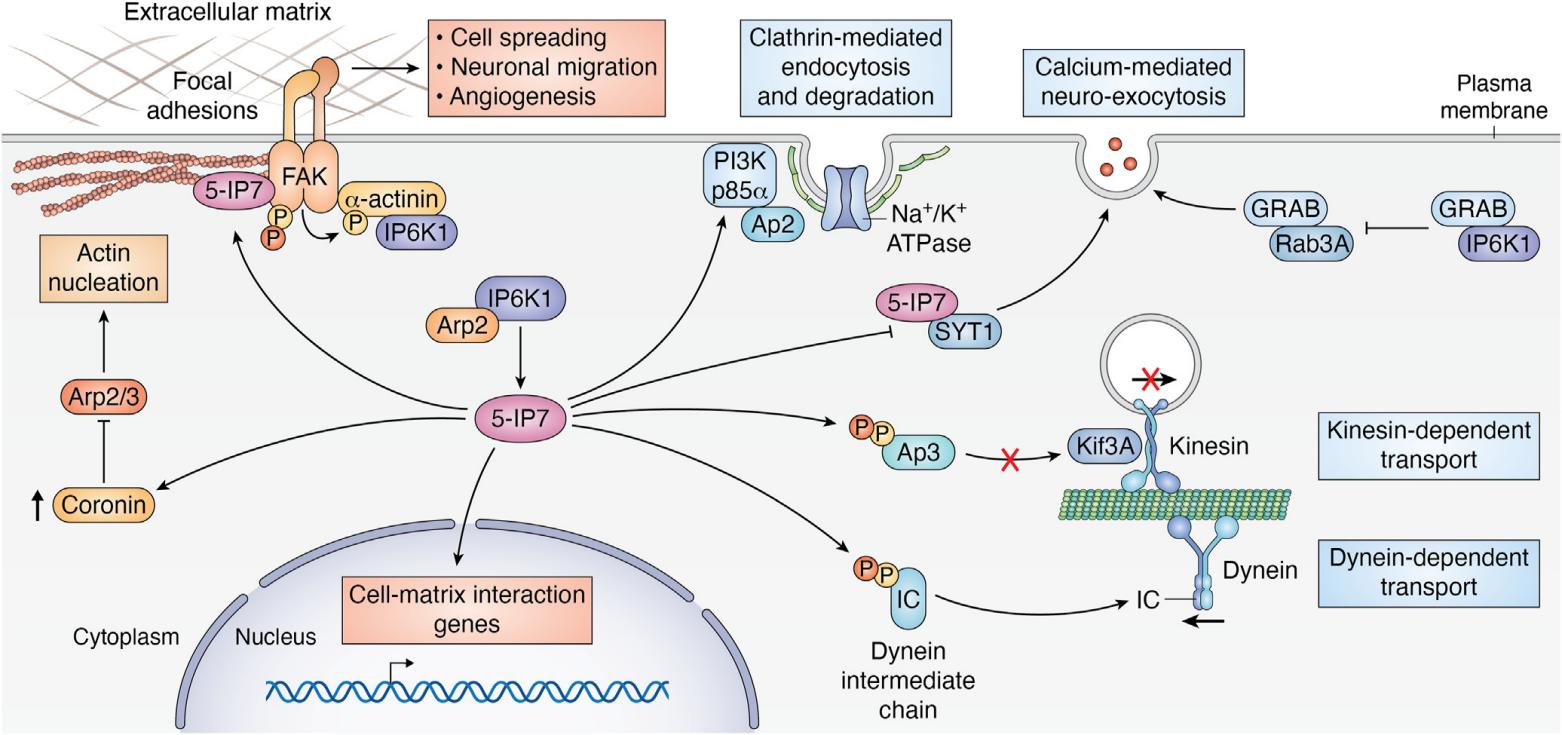
**Roles of IP6K1 in trafficking, migration, and cytoskeleton remodeling.** (1) Migration. IP6K1 binds α-actinin localized at focal adhesions, promoting its phosphorylation by focal adhesion kinase (FAK). 5-IP7 produced by IP6K1 binds to FAK and triggers its pyrophosphorylation and dimerization, thereby regulating interactions with the extracellular matrix, cell migration, and angiogenesis. In the nucleus, 5-IP7 enhances the expression of genes involved in cell-matrix interaction. (2) Cytoskeleton. 5-IP7, produced by the interaction of IP6K1 with actin-related protein 2 (Arp2), recruits the Arp2/3 inhibitor coronin and thereby disrupts actin filament production. (3) Trafficking. 5-IP7 triggers the pyrophosphorylation of dynein intermediate chain (IC), promoting dynein-dependent transport. 5-IP7 also triggers the pyrophosphorylation of adaptor protein 3 (AP3), blocking its interaction with the kinesin motor protein Kif3A and thus attenuating kinesin-dependent transport. Binding of 5-IP7 to the calcium sensor synaptotagmin 1 (SYT1) inhibits neurotransmitter exocytosis from neurons. Binding of IP6K1 to the guanine exchange factor GRAB prevents formation of the GRAB/Rab3A complex, thus inhibiting calcium-dependent neuroexocytosis. Binding of 5-IP7 to the phosphoinositide 3-kinase (PI3K) p85α subunit recruits adaptor protein 2 (AP2), triggering clathrin-mediated endocytosis and degradation of the sodium-potassium ATPase. 5-IP7, 5-diphosphoinositol pentakisphosphate; IP6K1, inositol hexakisphosphate kinase 1.

In mammals, the actin-myosin system plays a crucial role in facilitating the short-distance movement of endocytic and exocytotic vesicles close to the plasma membrane (15). Long-range transport across the cytoskeletal microtubules is driven by two classes of motor proteins: kinesins and dyneins (16). IPs negatively regulate the interaction between the kinesin motor protein (Kif3A) and its corresponding adaptor protein 3 (AP3). Specifically, 5-IP7-mediated pyrophosphorylation of AP3 reduces its interaction with Kif3A and prevents the exocytosis of viral particles from cells (17) (Fig. 2). Hamid, *et al.* (2023) further studied the role of IP6K1 in mediating AP3 pyrophosphorylation. IP6K1 was shown to interact with both AP3B1, the β subunit of AP3, and the catalytic α-subunit of CK2, a protein kinase that phosphorylates serine residues prior to pyrophosphorylation. This interaction results in the formation of AP3B1-IP6K1-CK2 complex allowing for synchronized and instantaneous prephosphorylation and pyrophosphorylation of the serine residue on the target protein (18). Further investigation is needed to verify whether IP6K1-mediated pyrophosphorylation of other target proteins is regulated by the same mechanism as in AP3. Additionally, three isoforms of the vesicular glutamate transporters are responsible for loading synaptic vesicles with glutamate. IP6K1 was shown to regulate the trafficking of vesicular glutamate transporter isoforms 1 and 2 *via* AP1/3 adaptors (19).

IPs also facilitate insulin exocytosis from pancreatic β-cells by expanding the readily releasable pool of insulin-containing vesicles and boosting insulin release from these vesicles (20) The involvement of IPs, and particularly 5-IP7, in the aforementioned myosin-actin and microtubule-dependent kinesin-driven processes, suggests a role for IP6Ks in trafficking. Chanduri *et al.* (2016) have shown that mammalian cells lacking IP6K1 and slime molds lacking the IP6K homolog, I6KA, develop a deficiency in dynein-dependent transport. In fact, IP6K1 produces a pool of 5-IP7s which mediates the pyrophosphorylation of the dynein intermediate chain (Fig. 2). This phosphorylation enhances the binding of intermediate chain to p150^Glued^ subunit of dynactin, interaction that is essential for dynein-dependent transport (21). Moreover, mammalian cells lacking IP6K1 exhibit several defects in dynein-dependent trafficking pathways, such as impaired endosomal sorting, vesicle movement, and Golgi maintenance (21).

IP6K1 through the production of 5-IP7 plays a key role in negatively regulating the activity of the Na^+^/K^+^-ATPase by triggering its endocytosis (22). The homeostatic turnover of Na^+^/K^+^ -ATPase depends on its coordinated endocytosis from, and insertion into, the plasma membrane. Under pathological conditions, the endocytosis of Na^+^/K^+^-ATPase requires the phosphorylation of its α subunit (23) and the interaction with phosphatidylinositol 3-kinase subunit p85α (24). Triggering the recruitment of adaptor protein 2 to activate clathrin-mediated endocytosis of the pump and drive its downstream degradation (25) (Fig. 2). Chin *et al.* (2020) have shown that 5-IP7, produced by IP6K1, binds with phosphatidylinositol 3-kinase p85α to facilitate its interaction with Na^+^/K^+^-ATPase. This recruits adaptor protein 2, triggering clathrin-mediated endocytosis and downstream proteasomal and lysosomal degradation of the pump (22).

Jadav R *et al.* performed a microarray-based gene expression analysis on mouse embryonic fibroblasts (MEFs) grown from Ip6k1−/− mice. Several genes involved in mediating cell-matrix interaction and downstream signaling were downregulated in cells lacking IP6K1 (26). Consequently, IP6K1-deficient cells exhibited reduced migration and invasion capabilities (26). The expression of active IP6K1 restores migration defects in IP6K1 knockout MEFs, indicating that the synthesis of 5-IP7 by IP6K1 is essential for promoting cell locomotion (26).

In mammalian cells, the actin-related protein two-thirds complex initiates the nucleation of actin filaments while focal adhesions link the actin cytoskeleton to the extracellular matrix (27). The focal adhesion kinase (FAK) plays a critical role in the turnover of focal adhesions, which is essential for cell migration and blood vessel formation. It was demonstrated that IP6K1 interacts with Arp2 and produces 5-IP7, leading to the recruitment of coronin, a negative regulator of the actin-related protein two-thirds complex. As well, IP6K1 generates an enriched pool of 5-IP7, which binds to FAK, activating it *via* promoting its dimerization and phosphorylation (27). The study also indicates that 5-IP7 modulates FAK, not only by binding to it but also through pyrophosphorylation (27). Further studies are required to determine the effect of FAK pyrophosphorylation by 5-IP7. Moreover, α-actinin plays a crucial role in the formation of actin bundles and acts as a linker, connecting actin to focal adhesions. This connection is essential for focal adhesion maturation (28). IP6K1 has a natural association with α-actinin and localizes to focal adhesions (29). Deletion of IP6K1 or inhibiting its catalytic activity in neuronal cells significantly reduces stress fiber formation and impairs cell migration and spreading. Thus, it was demonstrated that IP6K1 plays a vital role in neuronal migration by interacting with α-actinin and influencing the phosphorylation of both FAK and α-actinin through its product, 5-IP7 (29) (Fig. 2).

IP6K1 can play a complex role in blood vessel formation. Inhibiting IP6K1 or decreasing the levels of 5-IP7 increases glycolysis and activates AKT and AMPK signaling pathways, which activate angiogenesis. Depleting 5-IP7 from endothelial cells disrupts the turnover of focal adhesions, diminishing blood vessel formation (27). The exact biological roles of IP6K1 and 5-IP7 in angiogenesis are unclear and require further systematic investigation.

IP6K1 is involved in the formation of the blood–testis barrier, a testis-specific inter-Sertoli cell impermeable junction complex. It was demonstrated that IP6K1 knockout in mice testis causes blood–testis barrier disruption accompanied by transcriptional alteration of the tight junction protein claudin 3 and modifies the location of the gap junction protein connexin 43 (30). Bhat *et al.* (2024) showed that the loss of IP6K1 increases AKT/ERK and integrin signaling and augments the dephosphorylation of cofilin leading to the destabilization of cytoskeletal actin in Sertoli cells causing germ cell loss. Thus, the loss of IP6K1 attenuated germ cell adhesion, causing premature detachment of spermatids from the seminiferous epithelium into the epididymis.

Additionally, IP6K1 is involved in neuroexocytosis through enzyme-dependent and independent mechanisms. The guanine nucleotide exchange factor GRAB binds to Rab3A, a member of the Rab family of small GTPases, to form the GRAB/Rab3A complex required to trigger Ca^2+^ stimulated vesicles’ synaptic exocytosis (31). Synaptotagmin 1 (SYT1) is a Ca^2+^ sensor which mediates calcium-dependent quick release of neurotransmitters by promoting synaptic membrane fusion with vesicles (32). It was shown that both catalytically active and inactive IP6K1 forms inhibit the formation of GRAB/Rab3A complex by physically competing with GRAB for binding to Rab3A in PC12 cells (31). On the other hand, IP6K1 triggers the production of 5-IP7 which binds SYT1 to inhibit the calcium-mediated neuroexocytosis in hippocampal and PC12 neuronal cells (32) (Fig. 2). Similarly, IP6K1 deletion in acute hippocampal slices increased the probability of presynaptic exocytosis while decreasing short-term facilitation of synaptic vesicle fusion during endocytosis (33). Exocytosis and endocytosis were recovered when IP6K1 levels were fully restored in IP6K1-KO hippocampus cells. However, the expression of catalytically inactive IP6K1 restored short-term facilitation but failed to restore exocytosis (33). This suggests that IP6K1 plays both a catalytic role, *via* 5-IP7, and a noncatalytic role in the regulation of presynaptic events, inhibiting presynaptic vesicle exocytosis and stimulating presynaptic vesicle endocytosis at central synapses (33). The aforementioned observations lay the groundwork for a flourishing and innovative field of investigation, necessitating additional studies aimed not solely at elucidating the molecular mechanisms underlying the dynamic regulation of synaptic vesicle exocytosis–endocytosis by IP6K1 and 5-IP7 but also at formulating therapeutic strategies to modulate neurotransmitter release at central synapses.

In summary, through the production of the high energy potential 5-IP7 pool, IP6K1 plays a critical and dynamic role in regulating focal adhesion dynamics, actin cytoskeleton remodeling, microtubule trafficking, stress fiber formation, cellular migration, angiogenesis, and synaptic neurotransmitter exocytosis.

## Gene expression and DNA repair

**Figure 3:**
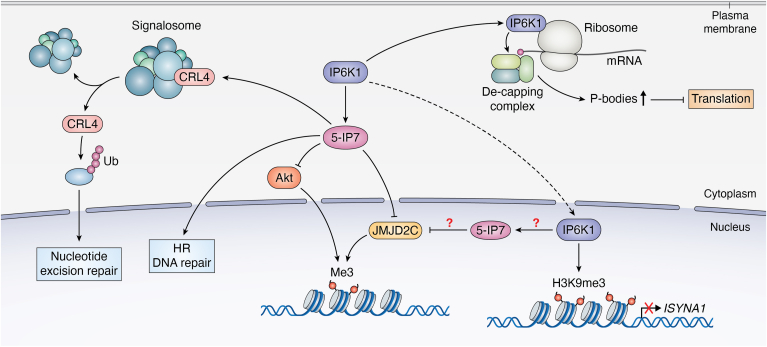
**Roles of IP6K1 in gene expression and DNA repair.** (1) DNA methylation. An IP6K1-mediated increase in 5-IP7 inhibits the histone lysine demethylase Jumonji domain containing 2C (JMJD2C). Inhibition of JMJD2C triggers the dissociation of JMJD2C from chromatin, thereby increasing trimethyl-histone H3 lysine 9 (H3K9me3) levels and inhibiting the transcription of target genes. 5-IP7 also inhibits protein kinase B (AKT), resulting in increased methylation of imprinted genes. (2) Recombination. 5-IP7 plays a crucial role in the progression and completion of homologous recombination (HR) DNA repair following DNA damage. (3) Gene expression. High levels of cell-membrane PA trigger the translocation of IP6K1 into the nucleus where it increases H3K9me3 levels in the *ISYNA1* promoter region. This results in inhibition of *ISYNA1* expression and decreased *de novo* synthesis of *myo*-inositol. (4) Translation. IP6K1 localized to the ribosome near the translation initiation complex can interact with the mRNA de-capping complex. This interaction decreases the number of mRNA de-capping proteins, consequently blocking mRNA translation and increasing the formation of P-bodies. (5) Protein degradation. Under stress conditions, the production of 5-IP7 by IP6K1 causes the dissociation and activation of CRL4 from the signalosome. Activated CRL4 ubiquitylates and degrades target proteins involved in nucleotide excision repair. 5-IP7, 5-diphosphoinositol pentakisphosphate; IP6K1, inositol hexakisphosphate kinase 1.

Epigenetic modifications of chromatin are considered a fundamental mechanism through which eukaryotic cells adjust their transcriptional response to developmental and environmental signals. Histones undergo various reversible posttranslational modifications, such as acetylation, phosphorylation, methylation, ubiquitylation, ADP ribosylation, and sumoylation (34). A study by Burton *et al.* (2013) indicated that the mammalian IP6K1 associates with chromatin and interacts with a recently identified histone lysine demethylase, Jumonji domain containing 2C (JMJD2C) (35). Reducing IP6K1 levels decreases 5-IP7 concentration, which is epigenetically translated to reduction in trimethyl-histone H3 lysine 9 (H3K9me3) levels. This decrease in H3K9me3 is a result of JMJD2C demethylase activity, which is activated in response to the decline of 5-IP7 levels (35). In contrast, the expression of IP6K1 generates JMJD2C dissociation from chromatin and increases H3K9me3 levels, dependent on IP6K1 catalytic activity (35). The association of IP6K1 to the chromatin as an endogenous regulator of JMJD2C and H3K9 methylation demonstrates that IPs play a crucial role in epigenetic histone modifications. In addition, IP6K1 was shown to repress expression of *ISYNA1*, a gene which encodes myo-inositol-3-P synthase, and to increase DNA methylation of the ISYNA1 promoter in MEF cells (36) (Fig. 3). This repression by IP6K1 may potentially involve several mechanisms, such as recruitment of transcription repressors, activation of DNA methylases, or impaired assembly of the transcription complex (36). The mechanisms could depend on IP6K1 catalytic activity or 5-IP7 levels and require further investigation.

AKT, also known as protein kinase B, is a well-known serine/threonine specific-protein kinase which plays significant roles in various cellular mechanisms, including growth, proliferation, glucose transport, migration, and DNA repair (37). It was also demonstrated that 5-IP7, the product of IP6K1, functions as an inhibitor of AKT (38). This inhibition, in turn, has a negative effect on the methylation of imprinted genes (39) (Fig. 3).

A study by Szijgyarto *et al.* (2011) suggests that in yeast cells, IPs, particularly 5-IP7, can influence the transcription of glycolytic genes by interacting with their transcription activators (GCR1 and GCR2) (40). 5-IP7 pyrophosphorylates one of the serine residues of GCR1, destabilizing its interaction with GCR2, and preventing its binding to the promoter regions of the glycolytic genes (40). Similarly, 5-IP7 was shown to pyrophosphorylate a serine residue within the PEST domain of MYC, triggering its polyubiquitination and degradation and, thus, regulating MYC turnover in mice (41). The effects are far-reaching, as MYC is a transcription factor that triggers cell growth and proliferation through regulating the expression of genes involved in glucose and amino acid metabolism, protein synthesis, cell cycle progression, and mitochondrial biogenesis (42).

Processing bodies or P-bodies are cytoplasmic ribonucleoprotein granules that store translationally repressed mRNA and are also enriched in proteins that mediate 5′-3′ mRNA degradation, including proteins responsible for mRNA de-adenylation, mRNA decapping, and 5′-3′ exoribonuclease activity (13). IP6K1, independent of its catalytic activity, binds to the translation initiation complex on ribosomes and interacts with the mRNA decapping complex, preventing the initiation of mRNA translation and leading to upregulation of P-body formation to store the resulting translationally repressed mRNAs (13). Thus, IP6K1 is a unique facilitator of proteome remodeling on the mRNA cap, tilting the balance toward translational repression over initiation, causing P-body formation (Fig. 3). In addition, the reduction of IP6K1 levels is associated with a decrease in the level of mRNA decapping proteins EDC4, DCP1A/B, and DCP2 (13). Further investigation is required to understand how IP6K1 regulates the levels of mRNA decapping proteins.

Several studies indicated a role for IP6K1 in DNA repair. The Cullin/signalosome complex mediates the process of protein ubiquitylation, which marks proteins for degradation. Under normal conditions, IP6K1 was shown to be associated with the Cullin/signalosome complex, which is thus held in an inactive state. CRL4 is a specific Cullin-RING ubiquitin ligase activated in response to DNA damage. Under stress conditions, such as UV radiation exposure, IP6K1 is stimulated to produce 5-IP7. This 5-IP7 then causes the dissociation of the CRL4/signalosome complex, leading to the activation of CRL4 (43). Consequently, target proteins are ubiquitylated and subsequently degraded, thus regulating nucleotide excision repair and cell death (43) (Fig. 3). Another study showed that the loss of IP synthesis by IP6K1 weakens homologous recombination (HR) repair in mammalian cells. IP6K1^−/−^ MEFs were capable of initiating HR but fail to complete it, leading to either cell death or the accumulation of chromosomal abnormalities (44). This indicates that IPs synthesized by IP6K1 are essential for regulating DNA repair. Therefore, IP6K1 is vital to protect genomic integrity in mammalian cells. Further investigation is required to understand exactly how IP6K1, through pyrophosphate synthesis, regulates HR signaling. This regulatory role might be due to the interaction of IPs with one or more proteins involved in the downstream process of HR repair. In this case, IPs might be pyrophosphorylating these proteins or physically binding to them and, thus, altering their activity (44).

## Metabolism

**Figure 4:**
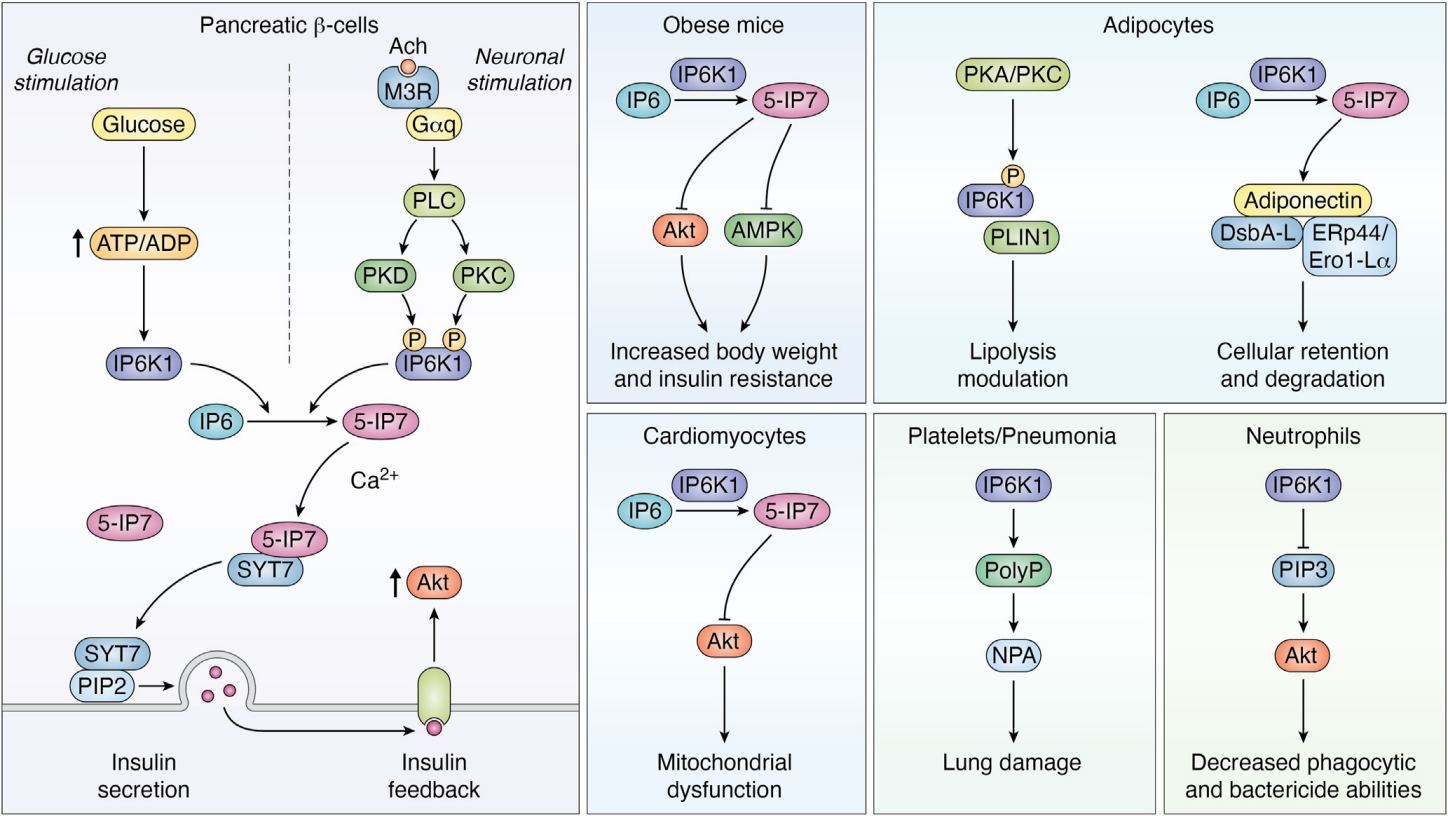
**Roles of IP6K1 in metabolism and immunity.** (1) Insulin secretion: High levels of glucose increase the ATP/ADP ratio in pancreatic β-cells; this change is sensed by IP6K1, which triggers the production of 5-IP7. Following neuronal stimulation, acetylcholine stimulates muscarinic M3 receptors (M3R) on β-cells triggering the sequential activation of the G-protein αq-subunit (G αq), PLC, PKC, and PKD to phosphorylate IP6K1. Phosphorylated IP6K1 is activated to produce high levels of 5-IP7. 5-IP7 binds Ca^2+^ ions and thereby frees synaptotagmin 7 (SYT7) to interact with PIP2, inducing insulin exocytosis. *Note: under basal conditions, 5-IP7 binds SYT7 and prevents its interaction with PIP2.* Secreted insulin binds to insulin receptors on β-cells, triggering the activation of AKT. (2) Obesity: 5-IP7 inhibits AKT and AMP-activated protein kinase (AMPK), causing an increase in body weight and insulin resistance in diet-induced obese mice. (3) Mitochondrial dysfunction: Inhibition of AKT by 5-IP7 in cardiomyocytes is linked to attenuation of mitochondrial function and biogenesis. (4) Lipolysis: In adipose tissue, the phosphorylation of IP6K1 on a PKA/PKC motif triggers its binding to the lipolytic regulator protein perilipin1 (PLIN1), thereby regulating lipolysis. 5-IP7 also binds to the adiponectin-DsbA-L complex and enhances its interaction with the Erp44/Ero1-Lα complex to trigger cellular retention and degradation of adiponectin. (5) Neutrophil defenses: In neutrophils, IP6K1 was shown to inhibit the PIP3-mediated membrane translocation of AKT, decreasing phagocytic and bactericidal capabilities. (6) Lung damage: During pneumonia infection, IP6K1 mediates the production of inorganic polyphosphates (polyP) in platelets. PolyP production leads to the formation of infection-induced neutrophil-platelet aggregates in the alveolar spaces, consequently contributing to lung damage. PIP2, phosphatidylinositol 4,5-bisphosphate; PIP3, phosphatidylinositol (3,4,5)-triphosphate; PKC, protein kinase C; PKD, protein kinase D; PLC, phospholipase C.

Several studies have discussed multiple roles for IP6K1 in metabolism, including roles in phosphate homeostasis, insulin sensitivity, bone integrity, lipolysis, and inositol synthesis. Maintaining phosphate homeostasis is essential for growth and survival due to its central role in most biochemical reactions within a living organism. In yeast, IPs have been identified as vital regulatory elements controlling phosphate homeostasis. Furthermore, IP6K1 and IP6K2 jointly control IP metabolism in mammalian cells (2). Through maintaining IP pools, IP6K1 and IP6K2 are involved in the physiological regulation of phosphate export through the *XPR1* phosphate exporter and other aspects of cellular phosphate homeostasis (2, 45). By modulating the glycolytic/mitochondrial metabolic ratio, IPs control ATP concentrations in MEF cells and yeast (40). During chronic kidney disease, phosphate renal excretion is alleviated, yielding high levels of bloodstream phosphates (hyperphosphatemia). Long-term inhibition of IP6Ks activity by SC-919 in chronic kidney disease rats increased kidney ATP, lowered hyperphosphatemia, and improved kidney functions (46). Thus, IP6K1 inhibition is a potential novel treatment strategy against hyperphosphatemia (46). Further studies are required to delineate the effect of specifically inhibiting IP6Ks in renal tissue rather than using a pan-inhibitor that targets all different tissue types (46).By sensing glucose levels and secreting insulin, pancreatic β-cells play essential roles in the control of glycemia. Disrupted energy metabolism in β-cells, particularly changes in basal ATP levels and ATP/ADP ratio in response to glucose, is linked to the development of type 2 diabetes in humans (47, 48). *myo*-Inositol derivatives play a key role in β-cell stimulus-secretion coupling, particularly IP6 and 5-IP7 (49). As mentioned before, 5-IP7 controls insulin exocytosis by β-cells (20). In fact, studies conducted on IP6K1 knockout mice (50) and mice treated with pan-IP6K inhibitor (51) have shown reduced serum insulin levels, suggesting that IP6Ks play a role in insulin secretion. IP6Ks have a high Km for ATP; thus, glucose-mediated changes in the ATP/ADP ratio might affect IP6Ks activity and regulate 5-IP7 levels (52). A study by Rajasekaran *et al.* (2018) has shown that the glucose-stimulated increase in ATP/ADP ratio causes an increase in IP6K1 activity, resulting in synthesis of the appropriate concentration of 5-IP7 and thereby ensuring optimal insulin release (53) (Fig. 4). Thus, IP6K1 plays a linker role between blood glucose concentration and 5-IP7-mediated insulin release, indicating that IP6K1 has a metabolic glucose sensing capacity. The study suggests that defective 5-IP7 generation could serve as the focal point that integrates multiple genetic and environmental factors, contributing to the commonly observed diabetes phenotype (disrupted insulin secretion) (53). One of the critical aspects of β-cell regulation and signal transduction is the feedback mechanism of insulin. Secreted insulin binds to its own receptors on β-cells, thus igniting a second signaling wave which includes the activation of AKT/PKB complex (54). This feedback loop by insulin is essential for β-cell function, particularly for the synthesis and exocytosis of insulin (54). Contrary to other insulin sensitive tissues (38, 55), 5-IP7 reduction *via* either IP6K1 knockdown or inhibition in β-cells decreased AKT/PKB activation (56). This reveals a novel interaction between IP6K1, through 5-IP7, and AKT/PKB in pancreatic β-cells (56). Two components control this unique situation. The first is 5-IP7-mediated insulin exocytosis (20), and the second is the autocrine insulin feedback on its β-cell receptors that activate AKT/PKB (54). Thus, decreasing 5-IP7 concentrations will reduce insulin secretion, which in turn will reduce AKT/PKB signaling through its feedback mechanism. This stimulatory effect of 5-IP7 on AKT/PKB through insulin feedback dominates over its inhibitory effect on AKT/PKB in other tissues (56) (Fig. 4).

On the other hand, insulin secretion from β-cells is not only regulated by glycemia levels, but it can be controlled by the autonomous nervous system through cholinergic and adrenergic neurotransmitters and their corresponding G protein–coupled receptors (57, 58). As discussed previously, IP6K1 regulates insulin secretion from β-cells by producing 5-IP7 (20). M3 muscarinic receptors (M3Rs) are G-protein–coupled acetylcholine receptors located on β cells, which sense acetylcholine released by parasympathetic nerve terminals in rodents and regulate insulin release (59). A study by Zhang *et al.* (2021) demonstrated that upon stimulation of the parasympathetic nerve, acetylcholine is released within the synaptic junction where it binds to M3R receptors triggering the dissociation of G_α/q11_. This G protein will activate the PLC-PKC-PKD axis, resulting in the phosphorylation of IP6K1 on specific serine residues (60). Activated IP6K1, *via* phosphorylation by both PKC and PKD enzymes, catalyzes the biogenesis of 5-IP7 (60). Synaptotagmin 7 (SYT7) is another isoform of Ca^2+^ sensing. Unlike the previously mentioned neuronal SYT1, SYT7 is located in pancreatic β-cells where it triggers insulin exocytosis (61). Under basal conditions, 5-IP7 and IP6 bind SYT7 and block its interaction with PIP_2_, thus blocking vesicle membrane fusion. Upon M3R stimulation, IP6K1 is activated to produce high levels of 5-IP7, triggering Ca^2+^ions to bind 5-IP7, thus freeing SYT7 to interact with PIP_2_, which induces insulin exocytosis (60) (Fig. 4). It has been suggested that the inositol polyphosphates support the clamping function of SYTs (62, 63), but the exact mechanism of clamping and unclamping is not fully known. How and when Ca^2+^ions trigger the 5-IP7 clamp release from SYT7 is not well understood and requires further investigation.

In addition to insulin secretion, IP6K1 was also shown to play a role in osteoporosis, aging, insulin resistance and obesity. IP6K1 knockout mice fed on high-fat diet maintained normal insulin sensitivity, within the insulin-responsive tissues, *via* sustaining activity of the insulin sensitizing protein kinase AKT (38, 55). In addition, IP6K1 inhibits AMPK (AMP stimulated protein kinase) activity, leading to a reduction in adipose tissue browning–mediated thermogenesis (64). Consequently, inhibiting IP6K1 reduces body weight and insulin resistance in diet induced obese mice through the activation of AMPK and AKT (51, 64) (Fig. 4). In fact, 5-IP7 is a known physiological inhibitor of AKT by binding to its PH-domain, preventing its translocation to the plasma membrane, and potently inhibiting its phosphorylation by PDK1 (38, 65). Moreover, IP6K1 knockout mice are protected from high-fat diet–induced weight gain, insulin resistance, and metabolic dysfunction (66, 67). Thus, IP6K1 is considered a target in obesity and type 2 diabetes. A study by Naufahu *et al.* (2018) showed that chemical inhibition of IP6K1 in C2C12 myotubes increased insulin signaling (68). Additionally, high intensity exercise decreased both mRNA and protein levels of IP6K1 in the muscles of prediabetic individuals, which may be linked to increased insulin sensitivity in these patients (68). However, how exactly high-intensity exercise decreases muscle IP6K1 and how this decrease is linked to insulin sensitivity are not fully clear. Further investigation is needed to decipher all signaling intermediates to complete the picture. Furthermore, IP6K1 was shown to regulate lipolysis by interacting with lipolytic regulator protein perilipin1 (69). Following its own phosphorylation at a PKA/PKC motif, IP6K1 binds to perilipin1 and modulates lipolysis (69) (Fig. 4). IP6K1 was also shown to play a vital role in regulating bone marrow mesenchymal stem cells in mice, in which IP6K1 is identified as a therapeutic target to avoid skeletal involution (continuous bone loss) in response to high-fat diet and obesity (70). A recent study has demonstrated that inhibiting IP6K1 in mice prevents obesity-induced bone loss (71). Based on these findings, the inhibition of IP6K1 using selective inhibitors provides a potentially efficient approach to manage obesity and type 2 diabetes (71). Moreover, through the inhibition of AKT, high levels of 5-IP7 in cardiomyocytes caused mitochondrial dysfunction, insulin resistance, and impaired glucose homeostasis in diabetic mice. However, decreasing 5-IP7 generation through inhibiting IP6K1 restores mitochondrial function and biogenesis (72). Thus, a potential interesting relationship exists between IP6K1/5-IP7 and mitochondrial function which requires additional research.

Additionally, while being the key enzyme for inositol-pyrophosphate generation in hepatocytes (38, 64), IP6K1 plays important role in hepatic metabolism and liver disease. Liver-specific IP6K1 knockout protected mice from diet-induced hepatic steatosis, fibrosis, and hyperglycemia. Furthermore, *IP6K1-KO* mice livers exhibited elevated levels of hepatic proteins associated with mitochondrial oxidative capacity including the hepatic form of mitochondrial uncoupling protein UCP2 (73). *IP6K1-KO* hepatocytes also displayed elevated glycolysis rate and increased mitochondrial oxygen consumption rate, indicating that IP6K1 modulates liver metabolism and hepatic respiration (73). RNA-Seq studies in *IP6K1-KO* liver have shown that coupled and uncoupled mitochondrial oxidative pathways were upregulated, while the gluconeogenic pathway was downregulated, confirming the improved metabolism observed in *IP6K1-KO* mice (73). In the liver, IP6K1 regulates various pathways that modulate energy metabolism, insulin sensitivity, and fibrogenesis. Moreover, protein *O*-GlcNAcylation is a posttranslational modification that results in the addition of O-GlcNAc to Ser/Thr residues on a target protein (74). The *O*-GlcNAcylation of proteins has been demonstrated to have a varying effect on hepatic metabolism in human liver, in which it inhibits insulin signaling, induces lipogenesis, and promotes nonalcoholic fatty liver disease (74). IP6K1 was shown to promote protein *O*-GlcNAcylation in human hepatocytes, and its inhibition may have a therapeutic effect *via* improving metabolic dysfunction during nonalcoholic fatty liver disease (73). Exactly how IP6K1 stimulates the O-GlcNAcylation of hepatic proteins is currently unknown and requires further investigation.

Inositol is a widely occurring six-carbon cyclitol that is crucial for the viability of eukaryotic cells. Inositol compounds play key roles in various physiological processes, including gene expression, trafficking, signal transduction, and membrane biogenesis. In mammalian cells, IP6K1 was demonstrated as a unique negative regulator of inositol synthesis (36). IP6K1 was shown to translocate to the nucleus after its interaction with plasma membrane phosphatidic acid (36, 75). In the nucleus, IP6K1 mediates downregulation of expression of *ISYNA1*, the gene that encodes the rate-limiting inositol biosynthetic enzyme myo-inositol-3-P synthase (75).

Moreover, IP6K1 was shown to be involved in spermatogenesis (76), and IP6K1 knockout in male mice leads to sterility (50) due to the absence of chromatoid bodies in IP6K1 knockout spermatids, which are ribonucleoprotein granules involved in control of mRNA translation, mRNA decay, and small RNA-mediated gene regulation (77). The absence of these chromatoid bodies leads to premature translation of the sperm-specific nuclear proteins TNP2 and PRM2, resulting in defective sperm differentiation and apoptosis of elongated spermatids (77). Additionally, IP6K1 knockout male mice demonstrate cell junction abnormalities, specifically in the junctions connecting the elongated spermatids to Sertoli cells in the seminiferous tubules (76). IP6K1 can be considered a predictable marker for male infertility since the knockout of IP6K1 affects several steps of sperm development (76). Further investigation is needed to understand how IP6K1 affects the different steps of spermatogenesis.

Adiponectin, a circulating protein produced by adipocytes, plays a role in providing cardiovascular and metabolic protection (78). Fu *et al.* (2024) have examined the role of IP6K1/5-IP7 in cardiac ischemia-reperfusion injury and found that inhibiting or deleting IP6K1 can reduce the severity of myocardial infarction *via* increasing adiponectin levels in plasma (79). In the endoplasmic reticulum of adipocytes, IP6K1 generates a pool of 5-IP7 that binds adiponectin and DsbA-L, a key chaperone for adiponectin biosynthesis. This binding enhances the interaction between the adiponectin/DsbA-L complex and the ERp44/Ero1-Lα complex. Subsequently, adiponectin is not secreted into the blood but rather retained and degraded in the adipocyte (79) (Fig. 4). However, inhibiting IP6K1 or depleting 5-IP7 disrupts the interaction between adiponectin/DsbA-L and ERp44/Ero1-Lα and releases adiponectin into the bloodstream. Thus, depleting 5-IP7 *via* inhibiting IP6K1 activity is a potential therapeutic approach to protect the heart from myocardial ischemia-reperfusion injury by boosting plasma adiponectin (79). Additionally, several studies indicate that IP6K1 through 5-IP7 plays crucial roles in the endoplasmic reticulum and Golgi apparatus (21, 80, 81). However, further studies are needed to explore their functions and molecular mechanisms in more detail.

## Immunity

In light of their wide-ranging physiological activities, the role of IPs has been studied in immune cells. However, modulating IP6K1 levels may have significantly variable outcomes depending on cell and tissue type. IP6K1-knockout neutrophils present augmented phosphatidylinositol (3,4,5)-triphosphate (PIP3)-mediated translocation of AKT into the plasma membrane (65). This translocation of AKT enhances the downstream signaling of PIP3 in murine neutrophils, which display increased phagocytic and bactericidal abilities and enhanced NADPH oxidase-mediated superoxide production (65). Similar phenotypes were replicated in human neutrophils when IP6K1 was pharmacologically inhibited (65). It was shown that IP6K1 mediates its negative regulatory effect in neutrophils through the synthesis of 5-IP7. 5-IP7 acts by blocking PIP3-mediated plasma membrane translocation of PH domain-containing proteins, including AKT and several other mediators (38, 65) (Fig. 4).

IP6K1 was targeted therapeutically to improve the neutrophil immune response against bacteria in mice. The pharmacological inhibition of IP6K1 competently improved host bacterial killing but reduced neutrophil accumulation in the lungs, reducing lung damage caused by both gram-positive and gram-negative bacterial pneumonia (82). During pneumonia infection, IP6K1 mediates the production of inorganic polyphosphate in platelets, which play a crucial role in the formation of infection-induced neutrophil–platelet aggregates in the alveolar spaces (Fig. 4). These aggregates build up pressure in the alveolar tissue causing lung damage. However, inhibiting IP6K1 reduced serum polyphosphate levels and led to a decrease in neutrophil–platelet aggregate formation (82). On the other hand, knocking out IP6K1 in mice weakened the immunity and accelerated tumor growth. In IP6K1 knockout mice, tumor cell growth was noticeably accelerated, and host survival was drastically reduced compared with wildtype mice (83). Further investigation indicated that IP6K1 deletion significantly lowered the number of immune suppressive myeloid cells and M1 polarized macrophages. Particularly, the number of antigen-presenting dendritic cells and CD8+ cytotoxic T lymphocytes was notably lowered near tumor tissue (83). In this manner, host IP6K1 acts as a tumor suppressor by fine-tuning various tumor-immune cell interactions. Further studies in different animal models are needed to elucidate the complex roles of IP6K1 in different host cells as an immune modulator and/or tumor suppressor. Modulating the levels and activity of IP6K1 through therapeutic drugs may help in improving immunity and controlling cancer spread, instilling hope for immune compromised and cancer patients.

## Conclusion

Being highly energetic dynamic molecules, IPs have gained considerable attention from the scientific community. This increasing interest in studying IPs has provided insights into their biological roles and associated deregulation problems. Among the various IPs, 5-IP7 is remarkably important for maintaining cell metabolic balance, ATP production, and phosphate homeostasis. The majority of 5-IP7 in mammalian cells is synthesized by the enzyme IP6K1. In this review, we summarized what is known about the roles played by IP6K1 in different cellular metabolic processes in mammals, with brief insights into future research directions. IP6K1 has been shown to influence several physiological processes such as trafficking, cytoskeletal remodeling, migration, gene expression, DNA repair, metabolism, and immunity. It is especially fascinating that this single molecule plays different regulatory roles in different cells or even in the same cell. IP6K1 can sense metabolic changes, activate, or inhibit enzymes, trigger cellular trafficking, and modulate epigenetics, all in a well-orchestrated manner.

As we move forward, several interesting future directions emerge from this review. The unknown roles of IP6K1 and 5-IP7 in angiogenesis requires systematic investigation, offering opportunities for innovative research. Elucidating the molecular mechanisms underlying the role of IP6K1 in synaptic vesicle dynamics and the potential therapeutic modulation of neurotransmitter release opens the avenue for transformative studies. The roles of IP6K1 in gene expression open avenues for further investigation. The gene repression mechanisms of IP6K1, its regulatory impact on mRNA de-capping proteins, and its role in HR signaling present challenging yet important opportunities for exploration. The unexplored roles of IP6K1/5-IP7 in mitochondrial function, endoplasmic reticulum, and Golgi apparatus beckon for further investigation. Unraveling the signaling intermediates behind high-intensity exercise-induced IP6K1 decrease and its linkage to insulin sensitivity requires comprehensive investigation. The role of calcium ions in triggering the dissociation of 5-IP7 from SYT7 during insulin release requires further exploration. The unknown facets of IP6K1 in spermatogenesis, its role in O-GlcNAcylation of hepatic proteins, and its roles as an immune modulator/tumor suppressor in diverse host cells necessitate focused studies.

Thus, while IP6K1 is a promising potential therapeutic target because of its role in many disease processes such as type 2-diabetes, obesity, mental disorders, metabolic dysfunction, and cancer, therapeutically targeting IP6K1 must consider the complexity of interactions in which it plays a role demanding further comprehensive investigations.

## Conflict of interest

The authors declare that they have no conflicts of interest with the contents of this article.
